# The relationship between parent's self-reported exposure to food marketing and child and parental purchasing and consumption outcomes in five countries: findings from the International Food Policy Study

**DOI:** 10.1017/jns.2023.88

**Published:** 2023-12-07

**Authors:** Julia Soares Guimarães, Elise Pauzé, Monique Potvin Kent, Simón Barquera, Alejandra Jáuregui, Gary Sacks, Lana Vanderlee, David Hammond

**Affiliations:** 1 School of Interdisciplinary Health Sciences, University of Ottawa, Ottawa, Canada; 2 School of Epidemiology and Public Health, University of Ottawa, Ottawa, Canada; 3 Center for Nutrition and Health Research, INSP, Cuernavaca, Mexico; 4 School of Health and Social Development, Deakin University, Victoria, Australia; 5 School of Nutrition, Université of Laval, Quebec City, Canada; 6 School of Public Health and Health Systems, University of Waterloo, Waterloo, Canada

**Keywords:** Children, Food environment, Food policy, Marketing, Parents

## Abstract

Food and beverage marketing influences children's food preferences and dietary intake. Children's diets are also heavily influenced by their family environment. The aim of this study was to assess the relationship between parent's self-reported exposure to unhealthy food marketing and a range of outcomes related to children's desire for and intake of unhealthy foods and beverages. The study also sought to examine whether these outcomes varied across different countries. The analysed data are from the International Food Policy Study and were collected in 2018 using an online survey. The sample included 5764 parents of children under 18, living in Australia, Canada, Mexico, the United Kingdom, or the United States. Binary logistic regressions assessed the link between the number of parental exposure locations and children's requests for and parental purchases of unhealthy foods. Generalized ordinal regression gauged the relationship between the number of exposure locations and children's consumption of such items. Interaction terms tested if these associations varied by country. Parental exposure to unhealthy food marketing was positively associated with parents reporting child purchase requests and purchase outcomes; and differed by country. Increased parental exposure to unhealthy food marketing was associated with slightly lower odds of children's weekly consumption of unhealthy foods, and this association varied by country. In conclusion, parental report of a greater range of food marketing exposure was associated with a range of outcomes that would increase children's exposure to unhealthy food products or their marketing. Governments should consider developing more comprehensive restrictions on the marketing of unhealthy foods.

## Introduction

Children's diets are heavily influenced by their family environment and parents play a critical role in shaping child dietary behaviours.^(1,2)^ A review published in 2018 showed that parental food habits are the most relevant factor influencing children's food choices.^(2)^ An extensive body of research has also shown that unhealthy food marketing influences child food preferences,^(3,4)^ immediate intake,^(3,4)^ request to parents,^(5)^ and obesity prevalence.^(6,7)^ Food marketing viewed on television, in digital media, outdoors, and in a variety of settings is predominantly for unhealthy products (e.g. ultra-processed food and beverages that are typically high in sugar, fat, and salt).^(8–10)^ Food marketing uses numerous advertising techniques that increase its persuasive power. A global benchmarking of television advertising conducted across twenty-two countries, found that amongst sixteen countries (including Canada, Australia, Mexico, among others), three out of ten food and beverage advertisements contained promotional characters, and two out of ten contained premium offers (e.g. competitions, games).^(9)^ Such advertising and the techniques used in this advertising constitute a stimulus for consumption^(4,11–13)^ and can have a negative impact on both child and adult health.^(7,13,14)^ A recent study found that a large proportion of adults in Australia, Canada, Mexico, the United States, and the United Kingdom reported being exposed to unhealthy food marketing on television and digital marketing, and to spokes-characters and licensed characters featured in food advertising.^(15)^

The behavioural and health outcomes stemming from exposure to food marketing are directly determined by the food environment in which people are immersed and that varies by country. Policies that restrict unhealthy food marketing can positively impact the food environment.^(16,17)^ Many jurisdictions have started to implement policies aimed at reducing children's exposure to food marketing, to improve their dietary behaviours, and to reduce and prevent child obesity. In Canada, the province of Quebec implemented regulations to protect children from all commercial advertising in a variety of media and child settings in 1980.^(18)^ The United Kingdom adopted legislation prohibiting food advertising targeting children on television in 2007, specifically targeting children under 16 years.^(19)^ Mexico, for its part, introduced regulations to limit food advertising targeted at children on television and in movie theatres, in 2015,^(20)^ and banned characters in unhealthy food packages since 2020.^(21)^ Other countries, such as the United States, most of Canada, and Australia, primarily rely on voluntary marketing restrictions developed by industry, which has been shown to have no impact.^(22–25)^ Importantly, most restrictions (government statutory regulations or industry self-regulatory codes) do not specifically aim to reduce adult exposure.

Though much research has demonstrated the relationship between food marketing and children's food intake and other outcomes, very few studies have examined the impact of parental exposure to food marketing on their own purchases or on children's health. Experimental studies have found that advertising and certain marketing techniques can influence the desirability, acceptability, and perceived healthfulness of unhealthy food products among parents,^(26,27)^ which could in turn influence their purchasing behaviour and their children's food intake. For instance, Pettigrew *et al.*^(5)^ found that parents with children aged 8–14 years exposed to television and digital energy-dense food advertisements viewed promoted products more positively, wanted to consume these products more, and thought they could be eaten more often when compared to parents who were only exposed to static pictures of the same products.^(27)^ Another study found that parental and child exposure to food advertisements were positively associated with child requests for unhealthy food products.^(5)^ Those findings are relevant because if advertising is able to portray unhealthy foods as more desirable or acceptable to parents then there is reason to think it might influence their purchases, which in turn might influence child intake. Little is known about whether parental exposure to unhealthy food marketing is associated with other behaviours like child purchase requests and actual purchases that may influence consumption of unhealthy products, such as fast food, sugary drinks, and snacks. However, such parental exposure has been discussed as a proxy for child exposure given that children and parents are frequently in the same environments during exposure.^(28)^

The objective of this study was to assess the relationship between parent's self-reported exposure to unhealthy food marketing and children's purchase requests, parental purchases, and children's intake of unhealthy food and beverages. The study also sought to examine whether these behavioural outcomes varied across Australia, Canada, Mexico, the United Kingdom (UK), and the United States (US). We hypothesized that parent's self-reported exposure to unhealthy food marketing would be positively associated with children's ownership of fast-food toys and branded merchandise, purchase requests for unhealthy food with licenced characters or spokes-characters, and parental purchasing of unhealthy foods with licensed characters or spokes-characters, and children's consumption of unhealthy food and beverages. We also hypothesized that these outcomes would likely vary by country.

## Methods

### Sampling

Data were from the 2018 wave of the International Food Policy Study (IFPS) adult survey, collected in November/December 2018. Online surveys were completed by 22 824 respondents from five countries: Australia, Canada, Mexico, the UK, and the US.^(29)^ The sample was recruited from the Nielsen Consumer Insights Global Panel using standardized recruitment sampling across countries. Nielsen drew random samples stratified for age and sex from the online panels in each country. The eligibility criteria included being 18 years of age or older and residing in the target country. All potential respondents were provided with information about the study and were asked to provide consent before participating.^(29)^ Ethics approval for the data collection was received by one of the University of Waterloo's Research Ethics Committees (ORE # 21460) and this secondary analysis received clearance from the University of Ottawa's research ethics board (ethics file number H-06-20-5886).

For this study, a subsample from the IFPS was used. Individuals with children under 18 years old were included in the study while those that reported not having children or having children 18 years old or older were excluded.

### Parental exposure to unhealthy food and beverage marketing

In the survey, parents were asked whether they had seen or heard any advertisements or promotions for ‘unhealthy foods’ in the last 30 days (30 d) by media and setting. These included television; radio; online/internet; mobile app/video game; social media; in a text message; magazine or newspaper; billboard or outdoor sign; on buses, bus stops, and other public transport; in movies or at the movie theatre; at school/campus; signs or displays in supermarkets, convenience stores, or restaurants; at a recreation/community centre; sports event, concerts, or community event; giveaways, samples, or special offers and other. In the survey, ‘unhealthy foods’ were described as ‘processed foods high in sugar, salt, or saturated fat, such as soda/pop, fast food, chips, sugary cereals, cookies, and chocolate bars.’ Respondents had the option to select as many locations or sources of exposure as they recalled. A summary exposure measure representing the number of locations parents reported being exposed to unhealthy food marketing was then calculated. This measure, which could range between 0 and 15, was treated as a continuous variable in the modelling analyses.^(30)^

### Purchasing requests outcomes and purchasing outcomes

Purchase request outcomes included child request of unhealthy food products with (i) licensed characters and (ii) spokes-characters in the last 30 d. Purchase outcomes included children's ownership of toys from fast-food restaurants (any toy that comes with the purchase of a fast-food meal), children's ownership of branded merchandise with logos for unhealthy food products (any item such as clothing, posters, and stickers that show the logo of an unhealthy food or drink brand), and parental purchase of unhealthy food products with (i) licensed characters and (ii) spokes-characters for their child in the last 30 d. For all outcomes, parents responded ‘yes’, ‘no’, ‘don't know’, or ‘refuse to answer’. The wording for these measures is available in Supplementary Table 1.

### Child consumption of unhealthy food and beverages

To assess children's weekly consumption of unhealthy food and beverages, parents were asked in a typical week how often their children ate or drank the following items: sugary drinks; fast food; sugary cereals; snacks such as chips; desserts, such as cakes, cookies, and ice cream; and candy or chocolate bars. Response options included more than once a day; every day; a few times a week, but not every day; once a week; only on special occasions; and never. To facilitate analyses and the interpretation of our results, responses were collapsed into three categories: high consumption (more than once a day, or every day), moderate consumption (a few times a week, but not every day, or once a week), and low consumption (only on special occasions, or never).

### Socio-demographic characteristics

A range of socio-demographic variables based on or adapted from national census measures in all countries were included in the study. Participants self-reported sex at birth (male or female) and age categorized as 18–29, 30–44, 45–59, and ≥60 years old. Education was classified as low, medium, or high following criteria specific to each country, according to the highest level of education completed.^(29)^ For instance in Canada, low meant less than a high school diploma or high school diploma, medium meant trade certificate/diploma/some university (below bachelor's level), and high meant bachelor's degree or more.^(29)^ For ethnicity, adapted census measures specific to each country were used, and participants were classified as ‘majority’ if they identified themselves as ‘white’, predominantly English-speaking, or non-indigenous (criteria terminology varied by country according to what was most appropriate). For perceived income adequacy, parents self-reported how easy it is for them to ‘make ends meet’ based on their monthly income (very difficult, difficult, neither easy nor difficult, easy, or very easy). Parents reported the age of each child under the age of 18 for up to 10 children. Data were coded such that three binary variables were created denoting the presence of any children under the age of 6, children aged 6–12 years, and children aged 13–17 years.

### Statistical analysis

Statistical analyses were conducted using Stata 14⋅2. Descriptive analyses (percentages and means) were used to describe the sample characteristics and variables of interest. Binary logistic regressions were used to evaluate the association between parental exposure and country with the dichotomous outcome variables (child ownership of toys from fast-food restaurants, child ownership of products with unhealthy food brand logos, child food purchase requests, and parental purchase of unhealthy food products with licensed characters or characters created by the company). After testing for proportional odds, it was observed that the effects of the country variable and exposure on ordinal outcome variables (children's weekly consumption of unhealthy food and beverages) were not consistent across the different thresholds. As a result, generalized ordinal logistic regression was used to assess the association between parental exposure and country with the ordinal outcome variables. Interaction terms were then included in models to test if the association between parental exposure and examined outcomes differed by country. While the cross-sectional design of this study limits our ability to infer a causal relationship between exposure and outcomes, examining whether these associations are consistent across different contexts will help to evaluate whether these associations are likely to be causal. Country-stratified binary logistic and ordinal regressions were then conducted to assess how the relationship between parental exposure and examined outcomes differed between countries. All models used Canada as the country of reference.

Models were adjusted for child age and parental sex at birth, age (categorical), perception of income adequacy, education, and ethnicity. The *P*-values from the regression models used the Benjamini–Hochberg procedure to control the false discovery rate which was applied to decrease the number of Type 1 errors (false positives). Data were weighted with post-stratification sample weights constructed using a raking algorithm with population estimates from the census in each country based on age group, sex, region, ethnicity (except in Canada), and education (except in Mexico).^(29)^ All analyses applied the complex survey analysis commands.

## Results

### Sample characteristics

A total of 22 824 participants responded to the IFPS surveys. Respondents that reported not having children or only having children 18 years old or older were excluded from the dataset (*n* 16 019 participants excluded). Also excluded from analyses were participants who responded ‘don't know’ or ‘refuse to answer’ to questions pertaining to child ownership of toys from fast-food restaurants and branded merchandise with logos for unhealthy food products, child request of unhealthy food products with licensed characters or spokes-characters, parental purchase of unhealthy food products with licensed characters or spokes-characters, or weekly consumption of unhealthy food and beverages; and those for whom level of education and ethnicity were coded as ‘not stated’, or perceived income adequacy level coded as ‘don't know’ or ‘refuse to answer’ (total of *n* 1041 participants excluded). The final analytical sample size was 5764 parents. Table 1 describes the sample characteristics, stratified by country (unweighted sample characteristics can be seen in Supplementary Table 2). The differences in socio-demographic characteristics and the prevalence of outcomes between parents in the analytical sample and those excluded were not statistically significant (data not shown). As such, we opted against imputing missing values in our analysis.

**Table 1.** Sample characteristics of parents from the IFPS (2018), weighted (*n* 5764)

	Total (*N* 5764)		Australia (*N* 819)		Canada (*N* 774)		Mexico (*N* 2028)		United Kingdom (*N* 1102)		United States (*N* 1041)	
Variable	% (n)	CI (95 %)	% (n)	CI (95 %)	% (n)	CI (95 %)	% (n)	CI (95 %)	% (n)	CI (95 %)	% (n)	CI (95 %)
Sex												
Male	46⋅3 (2769)	44⋅8–47⋅8	42⋅9 (358)	39⋅1–46⋅7	48⋅7 (361)	44⋅6–52⋅8	47⋅0 (1045)	44⋅4–49⋅6	45⋅6 (518)	42⋅1–49⋅2	46⋅8 (487)	43⋅0–50⋅5
Female	53⋅7 (2995)	52⋅2–55⋅2	57⋅1 (463)	53⋅3–60⋅9	51⋅3 (413)	47⋅2–55⋅4	53⋅0 (983)	50⋅4–55⋅6	54⋅4 (584)	50⋅8–57⋅9	53⋅2 (554)	49⋅5–57⋅0
Age												
18–29 years old	18⋅1 (1067)	17⋅0–19⋅4	17⋅0 (131)	14⋅3–20⋅2	15⋅7 (93)	12⋅6–19⋅5	20⋅9 (475)	19⋅0–22⋅9	17⋅6 (212)	15⋅1–20⋅5	16⋅3 (156)	13⋅7–19⋅3
30–44 years old	52⋅8 (3137)	51⋅2–54⋅3	57⋅1 (443)	53⋅3–60⋅8	55⋅2 (430)	51⋅0–59⋅3	48⋅7 (1081)	46⋅1–51⋅3	52⋅3 (573)	48⋅7–55⋅8	55⋅6 (610)	51⋅9–59⋅3
45–59 years old	26⋅0 (1381)	24⋅6–27⋅4	22⋅8 (211)	19⋅9–26⋅0	26⋅3 (227)	23⋅0–29⋅9	28⋅1 (445)	25⋅6–30⋅8	27⋅3 (279)	24⋅2–30⋅7	22⋅8 (219)	19⋅7–26⋅3
60+ years old	3⋅1 (179)	2⋅6–3⋅7	3⋅1 (34)	2⋅1–4⋅4	2⋅7 (24)	1⋅7–4⋅4	2⋅3 (27)	1⋅5–3⋅4	2⋅8 (38)	1⋅9–4⋅0	5⋅3 (56)	3⋅9–7⋅2
Education												
Low	30⋅2 (1041)	28⋅6–31⋅8	29⋅7 (165)	25⋅9–33⋅7	28⋅9 (124)	24⋅6–33⋅6	15⋅3 (274)	13⋅4–17⋅4	40⋅0 (219)	36⋅2–43⋅9	49⋅1 (259)	45⋅3–52⋅9
Medium	19⋅9 (1226)	18⋅8–21⋅1	33⋅2 (270)	29⋅7–36⋅9	36⋅4 (293)	32⋅7–40⋅3	12⋅0 (208)	10⋅3–13⋅9	24⋅0 (288)	21⋅3–26⋅9	7⋅0 (167)	6⋅0–8⋅3
High	49⋅9 (3497)	48⋅4–51⋅4	37⋅1 (384)	33⋅7–40⋅8	34⋅7 (357)	31⋅3–38⋅3	72⋅7 (1546)	70⋅3–75⋅1	36⋅0 (595)	33⋅0–39⋅1	43⋅9 (615)	40⋅4–47⋅4
Ethnicity												
Majority	75⋅0 (4555)	73⋅6–76⋅4	68⋅5 (653)	64⋅4–72⋅4	73⋅8 (567)	70⋅0–77⋅2	75⋅7 (1663)	73⋅0–78⋅2	84⋅6 (960)	81⋅6–87⋅2	69⋅9 (712)	66⋅5–73⋅2
Minority	25⋅0 (1209)	23⋅6–26⋅4	31⋅5 (166)	27⋅6–35⋅6	26⋅2 (143)	22⋅8–30⋅0	24⋅3 (365)	21⋅8–27⋅0	15⋅4 (142)	12⋅8–18⋅4	30⋅1 (329)	26⋅8–33⋅5
Income adequacy												
Very difficult	9⋅0 (428)	8⋅1–10⋅0	9⋅5 (69)	7⋅4–12⋅1	8⋅2 (53)	5⋅9–11⋅2	10⋅6 (176)	8⋅9–12⋅5	6⋅8 (61)	5⋅1–9⋅1	8⋅6 (69)	6⋅5–11⋅2
Difficult	23⋅9 (1297)	22⋅6–25⋅2	21⋅1 (157)	18⋅0–24⋅6	19⋅2 (139)	16⋅1–22⋅8	30⋅3 (592)	28⋅0–32⋅8	23⋅9 (239)	20⋅9–27⋅1	17⋅5 (170)	14⋅8–20⋅6
Neither easy nor difficult	34⋅2 (1968)	32⋅8–35⋅7	31⋅9 (261)	28⋅4–35⋅6	35⋅4 (267)	31⋅5–39⋅5	38⋅2 (789)	35⋅7–40⋅7	32⋅2 (347)	28⋅9–35⋅6	30⋅1 (304)	26⋅8–33⋅7
Easy	22⋅2 (1406)	21⋅0–23⋅4	24⋅8 (222)	21⋅7–28⋅2	24⋅2 (199)	21⋅0–27⋅7	17⋅3 (384)	15⋅5–19⋅2	25⋅6 (316)	22⋅6–28⋅7	24⋅0 (285)	21⋅1–27⋅1
Very easy	10⋅7 (665)	9⋅8–11⋅7	12⋅7 (110)	10⋅3–15⋅5	13⋅0 (116)	10⋅8–15⋅6	3⋅6 (87)	2⋅8–4⋅6	11⋅6 (139)	9⋅6–13⋅9	19⋅8 (213)	17⋅0–23⋅0

CI, confidence interval.

### Purchase request and purchase outcomes

**Fig. 1.**
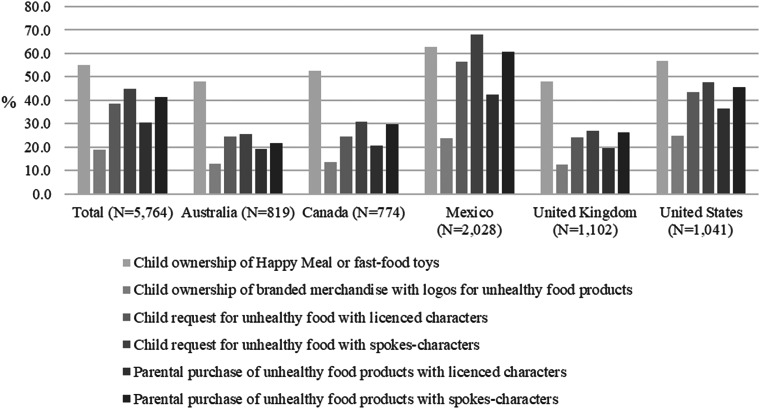
Purchase intent and purchase outcomes in the last 30 d, overall, and by country (*n* 5764).

As shown in Fig. 1, overall, more than half of parents (55⋅2 %) reported that their children owned toys from fast-food companies; 44⋅9 % reported that their children requested unhealthy food products with spokes-characters in the previous 30 d; in addition, 41⋅2 % of parents reported they purchased unhealthy food products with spokes-characters for their children. Amongst the five countries, Mexican parents consistently reported the highest levels of ownership of fast-food toys, child requests, and parental purchases of food products with spokes-characters (62⋅9, 68⋅1, and 60⋅5 %, respectively). The US had the highest percentage of parents reporting that their child owned branded merchandise from a food company (24⋅8 %).

### Child intake of unhealthy food and drink products

**Fig. 2.**
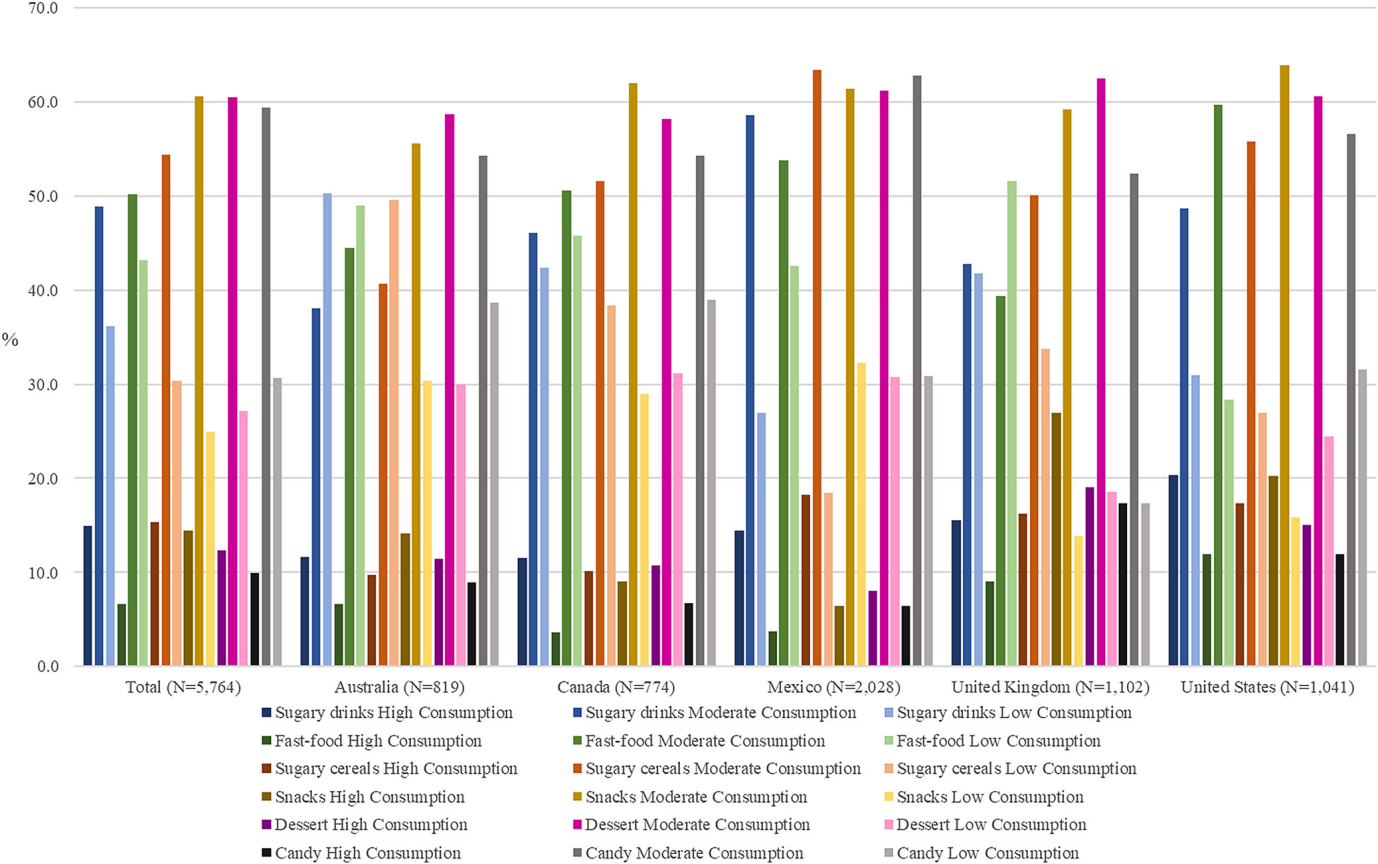
Child intake of unhealthy food and drink products during the week, overall, and by country (*n* 5764).

Weekly consumption of unhealthy food and beverages among participants’ children is shown in Fig. 2. Overall, 48⋅9–60⋅6 % of parents declared that children had a ‘moderate’ (a few times a week, but not every day and once a week) consumption of sugary drinks (48⋅9 %), fast-food (50⋅2 %), sugary cereals (54⋅4 %), snacks such as chips (60⋅6 %), desserts, such as cakes, cookies and ice cream (60⋅5 %), and candy or chocolate bars (59⋅4 %). The food consumed in ‘high’ amounts (more than once a day, or every day) most frequently within a country was snacks (26⋅9 % in the UK), followed by sugary drinks (20⋅4 % in the US), desserts (19⋅0 % in the UK), sugary cereals (18⋅2 % in Mexico), candy or chocolate bars (17⋅3 % in the UK), and fast food (12⋅0 % in the US).

### Country-level differences — purchase request and purchase outcomes

Overall, across the five countries, parents were exposed on average to marketing in 2⋅5 locations (sd = 2⋅9, Median = 2, IQR = 4), in Australia they were exposed to 2⋅0 locations (sd = 2⋅7, Median = 1, IQR = 3), in Canada to 1⋅7 locations (sd = 2⋅4, Median = 1, IQR = 3), in Mexico to 3⋅6 locations (sd = 3⋅1, Median = 3, IQR = 4), in the UK to 1⋅6 locations (sd = 2⋅2, Median = 1, IQR = 2), and in the US to 2⋅5 locations (sd = 2⋅8, Median = 2, IQR = 4).

The relationship between the number of locations where parents were exposed, country and purchase request, and purchase outcomes is presented in Table 2. In Australia, parents were less likely to report child ownership of fast-food toys (AOR = 0⋅760; CI = 0⋅602–0⋅958) than in Canada. In addition, child requests for unhealthy food with spokes-characters (AOR = 0⋅673; CI = 0⋅515–0⋅879), and purchase of unhealthy food products with spokes-characters (AOR = 0⋅598; CI = 0⋅454–0⋅788) were lower in Australia than in Canada. Conversely, compared to parents in Canada, those in the US and Mexico were more likely to report child ownership of branded merchandise with logos for unhealthy food products (AOR = 1⋅852; CI = 1⋅369–2⋅504 and AOR = 1⋅587; CI = 1⋅184–2⋅127, respectively), child requests for unhealthy food with licenced characters (AOR = 2⋅130; CI = 1⋅651–2⋅747 and AOR = 3⋅171; CI = 2⋅500–4⋅022, respectively), and spokes-characters (AOR = 1⋅778; CI = 1⋅386–2⋅280 and AOR = 3⋅800; CI = 3⋅007–4⋅803, respectively), and purchase of unhealthy food products with licenced characters (AOR = 2⋅122; CI = 1⋅612–2⋅794 and AOR = 2⋅416; CI = 1⋅866–3⋅127, respectively) and spokes-characters (AOR = 1⋅734; CI = 1⋅345–2⋅234 and AOR = 3⋅026; CI = 2⋅390–3⋅831, respectively).

**Table 2.** Binary logistic regression for child purchase request and parental purchase (*n* 5764)

	Child ownership of happy meal or fast-food toys		Child ownership of branded merchandise with logos for unhealthy food products		Child request for unhealthy food with licenced characters	
	AOR	CI (95 %)	AOR	CI (95 %)	AOR	CI (95 %)
Number of locations of parental exposure	1⋅100*	1⋅069–1⋅132	1⋅136*	1⋅106–1⋅166	1⋅206*	1⋅175–1⋅239
Country (Canada as reference)						
*Australia*	0⋅760*	0⋅602–0⋅958	0⋅865	0⋅614–1⋅217	0⋅896	0⋅681–1⋅178
*United Kingdom*	0⋅864	0⋅687–1⋅086	0⋅979	0⋅714–1⋅344	1⋅084	0⋅835–1⋅408
*United States*	1⋅155	0⋅911–1⋅466	1⋅852*	1⋅369–2⋅504	2⋅130*	1⋅651–2⋅747
*Mexico*	1⋅249	1⋅001–1⋅558	1⋅587*	1⋅184–2⋅127	3⋅171*	2⋅500–4⋅022

	Child request for unhealthy food with spokes-characters		Parental purchase of unhealthy food products with licenced characters		Parental purchase of unhealthy food products with spokes-characters	
	AOR	CI (95 %)	AOR	CI (95 %)	AOR	CI (95 %)
Number of locations of parental exposure	1⋅214*	1⋅179–1⋅249	1⋅125*	1⋅096–1⋅154	1⋅152*	1⋅121–1⋅184
Country (Canada as reference)						
*Australia*	0⋅673*	0⋅515–0⋅879	0⋅860	0⋅640–1⋅157	0⋅598*	0⋅454–0⋅788
*United Kingdom*	0⋅881	0⋅685–1⋅134	1⋅023	0⋅769–1⋅360	0⋅873	0⋅675–1⋅129
*United States*	1⋅778*	1⋅386–2⋅280	2⋅122*	1⋅612–2⋅794	1⋅734*	1⋅345–2⋅234
*Mexico*	3⋅800*	3⋅007–4⋅803	2⋅416*	1⋅866–3⋅127	3⋅026*	2⋅390–3⋅831

AOR, adjusted odds ratio; CI, confidence interval.

* *P*-value considered significant (*P* < 0⋅05) according to the Benjamini–Hochberg method; adjusted for the presence of children aged 5 years and under, 6–12 years, and 12–17 years, perceived income adequacy, and parental sex, age, ethnicity, and education.

### Relationship between exposure and examined outcomes

In the adjusted models with the full sample, parental exposure to a greater number of locations was associated with all purchase request and purchase outcomes (Table 2): child ownership of happy meal or fast-food toys (AOR = 1⋅100; CI = 1⋅069–1⋅132); child ownership of branded merchandise with logos for unhealthy food products (AOR = 1⋅136; CI = 1⋅106–1⋅166); child request for unhealthy food with licenced characters (AOR = 1⋅206; CI = 1⋅175–1⋅239); child request for unhealthy food with spokes-characters (AOR = 1⋅214; CI = 1⋅179–1⋅249); parental purchase of unhealthy food products with licenced characters (AOR = 1⋅125; CI = 1⋅096–1⋅154); and parental purchase of unhealthy food products with spokes-characters (AOR = 1⋅152; CI = 1⋅121–1⋅184). Table 3 presents the association between children's weekly consumption of unhealthy food, and number of locations of parental exposure, and differences between countries. Parental exposure to a greater number of locations was associated with lower odds of parents reporting children's moderate or high consumption of sugary drinks (AOR = 0⋅962; CI = 0⋅937–0⋅987), fast-food (AOR = 0⋅946; CI = 0⋅922–0⋅970), sugary cereals (AOR = 0⋅959; CI = 0⋅932–0⋅987), and candy (AOR = 0⋅972; CI = 0⋅947–0⋅998), compared to low consumption. Parental exposure to a greater number of locations also was associated with lower odds of parents reporting children's high consumption of fast-food (AOR = 0⋅937; CI = 0⋅903–0⋅973), sugary cereals (AOR = 0⋅958; CI = 0⋅931–0⋅985), snacks (AOR = 0⋅954; CI = 0⋅924–0⋅984), dessert (AOR = 0⋅958; CI = 0⋅928–0⋅990), and candy (AOR = 0⋅951; CI = 0⋅921–0⋅982), compared to low or moderate consumption.

**Table 3.** Generalized ordinal logistic regression for intake of unhealthy food and drink products during the week (*n* 5764)

	AOR	CI (95 %)	AOR	CI (95 %)	AOR	CI (95 %)
Predictor	Sugary drinks		Fast-food		Sugary cereals	
Moderate or high consumption (low consumption as reference)						
Number of locations where parents are exposed to unhealthy food marketing	0⋅962*	0⋅937–0⋅987	0⋅946*	0⋅922–0⋅970	0⋅959*	0⋅932–0⋅987
Country (Canada as reference)						
*Australia*	1⋅350*	1⋅072–1⋅699	1⋅108	0⋅881–1⋅393	1⋅614*	1⋅281–2⋅034
*United Kingdom*	0⋅985	0⋅788–1⋅232	1⋅262	1⋅011–1⋅576	0⋅826	0⋅658–1⋅037
*United States*	0⋅670*	0⋅527–0⋅852	0⋅510*	0⋅401–0⋅648	0⋅668*	0⋅522–0⋅855
*Mexico*	0⋅505*	0⋅405–0⋅630	0⋅975	0⋅789–1⋅205	0⋅406*	0⋅322–0⋅514
High consumption (low and moderate consumption as reference)						
Number of locations where parents are exposed to unhealthy food marketing	0⋅983	0⋅955–1⋅011	0⋅937*	0⋅903–0⋅973	0⋅958*	0⋅931–0⋅985
Country (Canada as reference)						
*Australia*	0⋅996	0⋅692–1⋅433	0⋅554*	0⋅325–0⋅944	1⋅111	0⋅736–1⋅678
*United Kingdom*	0⋅687*	0⋅450–0⋅964	0⋅340*	0⋅212–0⋅546	0⋅578*	0⋅395–0⋅847
*United States*	0⋅554*	0⋅397–0⋅774	0⋅336*	0⋅213–0⋅531	0⋅661*	0⋅453–0⋅964
*Mexico*	0⋅756	0⋅546–0⋅105	1⋅045	0⋅631–1⋅732	0⋅533*	0⋅375–0⋅757
	Snacks		Dessert		Candy	
Moderate or high consumption (low consumption as reference)						
Number of locations where parents are exposed to unhealthy food marketing						
Country (Canada as reference)	0⋅978	0⋅952–1⋅004	0⋅986	0⋅960–1⋅012	0⋅972*	0⋅947–0⋅998
*Australia*						
*United Kingdom*	1⋅004	0⋅787–1⋅279	0⋅912	0⋅711–1⋅168	0⋅967	0⋅767–1⋅218
*United States*	0⋅411*	0⋅314–0⋅540	0⋅520*	0⋅400–0⋅674	0⋅329*	0⋅256–0⋅424
*Mexico*	0⋅473*	0⋅362–0⋅617	0⋅740*	0⋅573–0⋅956	0⋅769*	0⋅605–0⋅978
High consumption (low and moderate consumption as reference)	1⋅137	0⋅908–1⋅423	1⋅069	0⋅852–1⋅340	0⋅709*	0⋅570–0⋅882
Number of locations where parents are exposed to unhealthy food marketing						
Country (Canada as reference)	0⋅954*	0⋅924–0⋅984	0⋅958*	0⋅928–0⋅990	0⋅951*	0⋅921–0⋅982
*Australia*						
*United Kingdom*	0⋅614*	0⋅422–0⋅893	0⋅945	0⋅659–1⋅355	0⋅733	0⋅472–1⋅138
*United States*	0⋅260*	0⋅186–0⋅365	0⋅498*	0⋅363–0⋅684	0⋅323*	0⋅218–0⋅480
*Mexico*	0⋅449*	0⋅316–0⋅640	0⋅759	0⋅544–1⋅058	0⋅614*	0⋅405–0⋅930

AOR, adjusted odds ratio; CI, confidence interval; p, *P*-value.

* *P*-value considered significant according to the Benjamini–Hochberg method; adjusted for the presence of children aged 5 years and under, 6–12 years, and 12–17 years, income adequacy, and parental sex, age, ethnicity, and education.

Models testing the interaction between country and parental exposure to unhealthy food marketing, and its association with the purchase request and purchase outcomes; and child's intake of unhealthy food products, are presented in the Supplementary Material, Tables 3 and 4. According to these models, the association between parental exposure and all examined outcomes varies between some countries. Country-stratified models are presented in Table 4. In these adjusted models, parental exposure to unhealthy food advertising was positively associated with all purchase requests and purchase outcomes in all five countries, except for child ownership of fast-food toys in Canada. However, the association between parent's self-reported exposure and children's weekly food intake differed by country in some instances. In Australia, the United Kingdom, and the United States, parents reporting exposure to unhealthy food marketing in a greater number of locations were less likely to report greater consumption of certain food categories among their children. In Canada and Mexico, the number of locations parents reported being exposed to unhealthy food marketing was not associated with children's food intake of any unhealthy food category.

**Table 4.** Adjusted odds ratios describing the association between the number of locations parents reported exposure to unhealthy food marketing and examined outcomes, stratified by country (*n* 5764)

	Canada	Australia	United Kingdom	United States	Mexico
Outcomes	AOR (95 %CI)	AOR (95 %CI)	AOR (95 %CI)	AOR (95 %CI)	AOR (95 %CI)
Child ownership of Happy Meal or fast-food toys	1⋅049 (0⋅978–1⋅125)	1⋅219 (1⋅137–1⋅308)*	1⋅134 (1⋅059–1⋅215)*	1⋅143 (1⋅075–1⋅215)*	1⋅053 (1⋅011–1⋅097)*
Child ownership of branded merchandise with logos for unhealthy food products	1⋅210 (1⋅114–1⋅315)*	1⋅274 (1⋅162–1⋅397)*	1⋅245 (1⋅154–1⋅344)*	1⋅127 (1⋅068–1⋅189)*	1⋅070 (1⋅032–1⋅108)*
Child request for unhealthy food with licenced characters	1⋅237 (1⋅139–1⋅344)*	1⋅313 (1⋅224–1⋅408)*	1⋅355 (1⋅258–1⋅459)*	1⋅201 (1⋅128–1⋅280)*	1⋅128 (1⋅087–1⋅170)*
Child request for unhealthy food with spokes-characters	1⋅267 (1⋅170–1⋅372)*	1⋅293 (1⋅201–1⋅393)*	1⋅323 (1⋅233–1⋅420)*	1⋅229 (1⋅153–1⋅310)*	1⋅127 (1⋅081–1⋅175)*
Parental purchase of unhealthy food products with licenced characters	1⋅140 (1⋅062–1⋅225)*	1⋅241 (1⋅154–1⋅335)*	1⋅263 (1⋅171–1⋅362)*	1⋅165 (1⋅097–1⋅237)*	1⋅051 (1⋅016–1⋅088)*
Parental purchase of unhealthy food products with spokes-characters	1⋅177 (1⋅091–1⋅271)*	1⋅218 (1⋅131–1⋅311)*	1⋅255 (1⋅163–1⋅354)*	1⋅184 (1⋅114–1⋅259)*	1⋅089 (1⋅047–1⋅132)*
Children's weekly consumption					
*Moderate or high consumption (low consumption as reference)*					
Sugary drinks					
Fast-food	0⋅927 (0⋅861–0⋅998)	0⋅939 (0⋅883–1⋅000)	0⋅892 (0⋅831–0⋅957)*	0⋅970 (0⋅912–1⋅030)	0⋅990 (0⋅948–1⋅033)
Sugary cereals	0⋅935 (0⋅873–1⋅002)	0⋅928 (0⋅876–0⋅984)*	0⋅874 (0⋅815–0⋅938)*	0⋅892 (0⋅825–0⋅963)*	0⋅981 (0⋅946–1⋅018)
Snacks	0⋅948 (0⋅886–1⋅014)	0⋅934 (0⋅875–0⋅997)	0⋅889 (0⋅835–0⋅946)*	0⋅978 (0⋅918–1⋅014)	0⋅970 (0⋅922–1⋅021)
Dessert	0⋅958 (0⋅891–1⋅031)	0⋅901 (0⋅831–0⋅976)*	0⋅905 (0⋅842–0⋅973)*	0⋅959 (0⋅903–1⋅019)	1⋅014 (0⋅977–1⋅053)
Candy	0⋅957 (0⋅886–1⋅032)	0⋅937 (0⋅872–1⋅008)	0⋅992 (0⋅914–1⋅077)	0⋅977 (0⋅916–1⋅043)	1⋅001 (0⋅962–1⋅040)
*High consumption (low and moderate consumption as reference)*	0⋅947 (0⋅883–1⋅016)	0⋅930 (0⋅865–1⋅001)	1⋅016 (0⋅943–1⋅093)	0⋅939 (0⋅886–0⋅996)*	0⋅996 (0⋅959–1⋅035)
Sugary drinks					
Fast-food	0⋅963 (0⋅871–1⋅066)	0⋅937 (0⋅871–1⋅001)	0⋅947 (0⋅882–1⋅017)	0⋅933 (0⋅882–0⋅987)*	1⋅029 (0⋅980–1⋅080)
Sugary cereals	0⋅934 (0⋅827–1⋅054)	0⋅927 (0⋅854–1⋅005)	0⋅843 (0⋅766–0⋅928)*	0⋅888 (0⋅819–0⋅965)*	1⋅000 (0⋅919–1⋅088)
Snacks	0⋅929 (0⋅843–1⋅025)	0⋅929 (0⋅859–1⋅005)	0⋅889 (0⋅835–0⋅946)*	0⋅944 (0⋅886–1⋅005)	0⋅992 (0⋅950–1⋅035)
Dessert	0⋅964 (0⋅908–1⋅023)	0⋅915 (0⋅854–0⋅980)*	0⋅858 (0⋅792–0⋅931)*	0⋅894 (0⋅839–0⋅953)*	1⋅049 (0⋅984–1⋅118)
Candy	0⋅976 (0⋅887–1⋅074)	0⋅941 (0⋅881–1⋅005)	0⋅898 (0⋅831–0⋅970)*	0⋅896 (0⋅824–0⋅975)*	1⋅018 (0⋅967–1⋅071)

AOR, adjusted odds ratio; CI, confidence interval.

* *P*-value (*P* < 0⋅05) considered significant according to the Benjamini–Hochberg method; models included the number of locations of parental exposure and were adjusted for the presence of children aged 5 years and under, 6–12 years, and 12–17 years, perceived income adequacy, and parental sex, age, ethnicity, and education.

## Discussion

### Summary of findings

Over half of parents across all countries reported that their children owned toys from fast-food companies. Mexico and the US had a high prevalence of parents reporting child purchase request and parental purchase outcomes. Parents in Mexico and the US also reported the highest weekly consumption of unhealthy food and beverage products among their children. The data also revealed that in each country, the number of locations where parents reported being exposed to unhealthy food marketing was positively associated with child purchase request and parental purchase outcomes. In addition, our results showed that parental exposure to a greater number of locations was associated with slightly lower odds of children's weekly consumption of unhealthy foods in some countries while no association was found in others. These findings were consistent with our initial hypotheses, with the exception of parent's self-reported exposure to unhealthy food marketing being negatively or not at all associated with children's consumption of unhealthy food and beverages.

Though this is the first study to our knowledge to examine the association between parent's exposure alone on child behavioural outcomes, a significant body of evidence has shown that child exposure to food and beverage marketing and child food intake have a causal association.^(31)^ A systematic review of twenty-two articles and a meta-analysis of eighteen papers published in 2016 concluded that food advertising influences children's food intake.^(12)^ Moreover, some research has shed light on the influence of food marketing and packaging on parental choices, purchase intentions, and product and brand perceptions. For instance, nutrition-related claims on beverages can lead parents to choose less healthy options for their children and misinterpret the healthiness of fruit drinks.^(32)^ Another paper demonstrates that food packages featuring smiling faces activate child-related thoughts in adults, leading to expectations of happiness and increased likelihood of purchasing the product for children.^(33)^ Additionally, adults perceive cereals with cartoon characters as less suitable.^(34)^ Lastly, one study highlighted the impact of conventional confectionery advertisements on parents, showing that exposure increases preference for the advertised product, distorts perceptions of healthiness and sugar content, and boosts brand attitude.^(35)^

Our findings did not demonstrate a positive association between parental exposure to unhealthy food marketing and child food intake. In fact, in some countries, parents reporting exposure to more marketing locations were less likely to report higher child consumption of unhealthy food categories. There are several reasons why a positive association might not have been observed as expected. Firstly, food choices are influenced by a multitude of factors, including cultural, social, economic, and individual preferences.^(36)^ Unhealthy food marketing is just one of many influences on food choices, and its impact may vary depending on the context and individual characteristics.^(37)^ Additionally, diverse parenting styles play a role, as some parents may actively promote healthier diets, mitigating the influence of marketing on their children's food intake.^(38)^ Moreover, the complex determinants of diet quality factors make it challenging to isolate the specific influence of marketing on child food intake.^(39)^ These unexpected findings may also be related to our sub-optimal measures. First, it's essential to acknowledge that our reliance on self-reported exposure introduces a potential bias, as individuals might underreport their exposure to unhealthy food and beverage marketing due to memory lapses or social desirability. Furthermore, the omission of assessing the intensity and frequency of exposure leaves us with an incomplete understanding of the true impact. The inherent disparity between the broad nature of our exposure measure, which encompasses a wide range of unhealthy food marketing, and the specific categorization of child food consumption variables (e.g. candy, snacks) could potentially confound the association we are trying to establish. Moreover, assuming that exposure's influence remains consistent across various settings like television and outdoor environments might oversimplify the complex dynamics at play. Additionally, our measure assumes a homogenous level of exposure influence across all children, overlooking the intricate variations in dietary habits within families with multiple children. This could be particularly problematic when one child's consumption drastically differs from another's, leading to inaccurate parental responses in our consumption measure. In light of these limitations, it is imperative to interpret our findings cautiously and consider avenues for refining our measurement strategies to better capture the nuanced relationship between unhealthy food marketing exposure and child consumption patterns. Nevertheless, our findings did show an association between parental marketing exposure with child purchase request and parental purchase of unhealthy food, both precursors to child consumption. As a result, parental exposure may potentially be considered a proxy for child exposure as has been previously concluded.^(28)^

Our findings also demonstrated that purchase requests, parental purchase, and children's consumption of unhealthy foods and beverages vary by country. When compared to Canada, Mexico and the US had a higher probability of child request and parental purchase of unhealthy food products with licensed characters and spokes-characters. While it is important to note that individual factors and cultural contexts can contribute to differences in food purchasing behaviours, several other factors may help explain why these results were observed in those countries. A contributing factor is the pervasive presence of unhealthy food marketing and advertising targeted towards children in Mexico^(9,40)^ and the US.^(41)^ In 2015, Mexico, implemented a set of regulations aimed at limiting unhealthy food advertising to children under age 12 both in television and movie theatres.^(20)^ Research evaluating Mexico's policy, however, has found that child exposure to unhealthy food products has not been reduced, as advertisers are shifting their marketing techniques towards the general public and families,^(42,43)^ and shifting the focus to other media.^(11,44,45)^ Furthermore, Mexican regulations, at the time of this study, did not likely lead to substantial improvements in children's consumption as the nutrition quality standards in the Mexican food marketing regulations are based on an industry derived criteria, which are much weaker than the standards applied in other countries, such as in the UK.^(43)^ Previous IFPS studies have similarly shown that Mexico is the country with the highest self-reported exposure to marketing strategies, and the most unfavourable food environment^(15,46–48)^ It should be noted, however, that Mexico revised its marketing restrictions in 2020 and has since banned cartoon characters on product packaging and has adopted more stringent nutritional standards.^(21)^ Moreover, our results reflect the scenario in Mexico in 2018, before the changes in marketing regulations in the country in 2020. Additionally, despite the well-established evidence, there is a concerning lack of regulations in the United States to curtail the promotion of unhealthy foods through advertising.^(9)^ This regulatory gap allows for the widespread marketing of products that contribute to poor dietary habits and undermines efforts to promote healthier food choices.^(16)^

Other factors influencing the high purchase of unhealthy food products in Mexico and the United States are socioeconomic disparities, cultural norms, and the broader food system context. Research suggests that lower-income individuals and families may have limited access to affordable, nutritious food options, leading to a higher reliance on cheaper, energy-dense, and nutrient-poor foods.^(49–51)^ This disparity in food access and affordability can contribute to the consumption of unhealthy food products.^(49)^ These factors influence the types of foods that are accessible, affordable, and prominently available in the food environment.^(51)^ In both countries, the food system context can contribute to the abundance and easy availability of unhealthy food options. Recent and future actions and regulations seeking to improve the food environment in the five countries examined appear warranted.^(16,21)^ Governments need to continue to monitor the food environment in their countries so that effective policies can be developed that foster health eating habits among children.

### Strengths and weaknesses

Our research is innovative in that it compared the relationship between parents’ self-reported exposure to food marketing and child and parental behavioural outcomes in a diverse international sample of adults. The strengths and limitations of the IFPS design have been reported elsewhere.^(52)^ In particular, this study is subject to limitations common to survey research, as previously mentioned in the discussion. The self-reporting of marketing exposure in a period of 30 d could introduce recall bias and likely only capture a small portion of actual exposure.^(53)^ The survey also did not measure the extent of marketing exposure such as the number of times a person was exposed to a particular media/setting. Additionally, the use of an online survey might have also created coverage bias, since in Mexico 35 % of households do not have internet access.^(54)^ In addition, this is a cross-sectional analysis and cannot be used to infer causal associations between predictors of interest and outcomes. The recruitment method used non-probability-based sampling; therefore, the findings do not provide nationally representative estimates. For example, although the data were weighted by age group, sex, region, ethnicity (except in Canada), and education (except in Mexico), the Mexican sample had notably higher education levels than census estimates. The survey questionnaire also does not allow us to establish a direct association between food marketing policies implemented (or not) in these countries and the outcomes evaluated. Another relevant weakness is that the variable of child ownership of branded merchandise with logos of unhealthy food products did not specify if those products were purchased or free merchandise received by children or parents. Also, we did not look into the exposure to any specific marketing techniques that parents were exposed to (e.g. use of animation/cartoon, giveaways, child-appealing packaging, etc.), which would most likely influence outcomes.^(55)^ Future studies could improve these findings by exploring a causal association of parental exposure to food marketing and child and parental purchasing and consumption outcomes.

## Conclusion

We found that higher parental reporting of exposure to food and beverage marketing was associated with a range of outcomes that would increase children's exposure to unhealthy food products or their marketing. The former also increased the likelihood that children owned toys from fast-food restaurants and branded merchandise with logos for unhealthy food products, that children would request unhealthy food products with licensed characters or spokes-characters, and that parents would purchase those same products. Parents may be unaware of the impact of unhealthy food marketing on their own purchasing behaviours for their children; however, such behaviours have a potentially large impact on child health. Governments should consider monitoring unhealthy food marketing to parents given the potential health impact this exposure has on children and their health. We also call for more comprehensive restrictions on the marketing of unhealthy foods such that multiple media channels and marketing techniques are included, and restrictions are based on stringent nutrient profiling of products.

## List of abbreviations

AOR, adjusted odds ratio
IFPS, International Food Policy Study
IQR, interquartile range (IQR)
NCD, noncommunicable diseases
SD, standard deviations
UK, United Kingdom
US, United States

## Supporting information

Soares Guimarães et al. supplementary material Soares Guimarães et al. supplementary material

## Supplementary material

The supplementary material for this article can be found at https://doi.org/10.1017/jns.2023.88
