# Supplementary material for: The relationship between parent's self-reported exposure to food marketing and child and parental purchasing and consumption outcomes in five countries: findings from the International Food Policy Study
Source: J Nutr Sci. 2023 Dec 7;12:e123. doi: 10.1017/jns.2023.88 (PMC10753451; doi:10.1017/jns.2023.88)
Supplement: Soares Guimarães et al. supplementary material [file S2048679023000885sup001.docx]

**Supplementary material**

Supplementary table 1. Outcomes related to purchase and purchase request.

|  | **Outcome** | **Further description** | **Question on survey** | **Possible answers** |
| --- | --- | --- | --- | --- |
| **Purchase request** | Child request of unhealthy food products with licensed characters. | Unhealthy foods include processed foods high in sugar, salt, or saturated fat, such as soda/pop, fast food, chips, sugary cereals, cookies and chocolate bars. | In the last 30 days, have your children asked you to buy any unhealthy food or drinks with characters from movies or TV (e.g., Star Wars, Disney characters)? | - Yes - No - Don’t know - Refuse to answer |
|  | Child request of unhealthy food products with spokes-characters. |  | In the last 30 days, have your children asked you to buy any unhealthy food or drinks with characters created by food companies (e.g., Tony the Tiger, Ronald McDonald)? |  |
| **Purchase** | Children’s ownership of toys from fast-food restaurants. | Toys from fast-food restaurants are any toy that comes with the purchase of a fast-food meal. | Do any of your children own any ‘Happy Meal’ toys or other toys from fast-food restaurants? |  |
|  | Children’s ownership of branded merchandise with logos for unhealthy food products. | Merchandise is any item such as clothing, posters and stickers that show the logo of an unhealthy food or drink brand, but not necessarily from the purchase of such item. | Do any of your children own any clothing, posters, stickers, or other products that show a logo or brand of unhealthy food or drinks? |  |
|  | Parental purchase of unhealthy food products with licensed characters for their child | Unhealthy foods include processed foods high in sugar, salt, or saturated fat, such as soda/pop, fast food, chips, sugary cereals, cookies and chocolate bars. | In the last 30 days, did you buy your children any unhealthy food or drinks with characters from movies or TV (e.g., Star Wars, Disney characters)? |  |
|  | Parental purchase of unhealthy food products with spokes-characters for their child |  | In the last 30 days, did you buy your children any unhealthy food or drinks with characters created by food companies (e.g., Tony the Tiger, Ronald McDonald)? |  |
| Source: Hammond, D. International Food Policy Study: All Country Codebook – 2018 Survey (Wave 2). University Of Waterloo. August 2019. | | | | |

Supplementary table 2. Sample characteristics of parents from the IFPS (2018), unweighted. (n=5,764).

| **Variable** | **Total (N=5,764)** | | **Australia (N=819)** | | **Canada (N=774)** | | **Mexico (N=2,028)** | | **United Kingdom (N=1,102)** | | **United States (N=1,041)** | |
| --- | --- | --- | --- | --- | --- | --- | --- | --- | --- | --- | --- | --- |
|  | **% (n)** | **CI (95%)** | **% (n)** | **CI (95%)** | **% (n)** | **CI (95%)** | **% (n)** | **CI (95%)** | **% (n)** | **CI (95%)** | **% (n)** | **CI (95%)** |
| Sex |  |  |  |  |  |  |  |  |  |  |  |  |
| Male | 48.0 (2,769) | 46.8 - 49.3 | 43.7 (358) | 40.3 - 47.1 | 46.6 (361) | 43.1 - 50.2 | 51.5 (1,045) | 49.4 - 53.7 | 47.0 (518) | 44.1 - 50.0 | 46.8 (487) | 43.8 - 49.8 |
| Female | 52.0 (2,995) | 50.7 - 53.2 | 56.3 (463) | 52.9 - 59.7 | 53.4 (413) | 49.8 - 56.9 | 48.5 (983) | 46.3 - 50.6 | 53.0 (584) | 50.0 - 55.9 | 53.2 (554) | 50.2 - 56.2 |
| Age |  |  |  |  |  |  |  |  |  |  |  |  |
| 18-29 years old | 18.5 (1,067) | 17.5 - 19.5 | 16.0 (131) | 13.6 - 18.7 | 12.0 (93) | 9.9 - 14.5 | 23.4 (475) | 21.6 - 25.3 | 19.2 (212) | 17.0 - 21.7 | 15.0 (156) | 12.9 - 17.3 |
| 30-44 years old | 54.4 (3,137) | 53.1 - 55.7 | 54.1 (443) | 50.7 - 57.5 | 55.6 (430) | 52.0 - 59.0 | 53.3 (1,081) | 51.1 - 55.5 | 52.0 (573) | 49.0 - 54.9 | 58.6 (610) | 55.6 - 61.6 |
| 45-59 years old | 24.0 (1,381) | 22.9 - 25.1 | 25.8 (211) | 22.9 - 28.9 | 29.3 (227) | 26.2 - 32.6 | 21.9 (445) | 20.2 - 23.8 | 25.3 (279) | 22.8 - 28.0 | 21.0 (219) | 18.7 - 23.6 |
| 60+ years old | 3.1 (179) | 2.7 - 3.6 | 4.2 (34) | 3.0 - 5.8 | 3.1 (24) | 2.1 - 4.6 | 1.3 (27) | 0.9 - 1.9 | 3.4 (38) | 2.5 - 4.7 | 5.4 (56) | 4.2 - 6.9 |
| Education |  |  |  |  |  |  |  |  |  |  |  |  |
| Low | 18.1 (1,041) | 17.1 - 19.1 | 20.1 (165) | 17.5 - 23.0 | 16.0 (124) | 13.6 - 18.8 | 13.5 (274) | 12.1 - 15.1 | 19.9 (219) | 17.6 - 22.3 | 24.9 (259) | 22.3 - 27.6 |
| Medium | 21.3 (1,226) | 20.2 - 22.3 | 33.0 (270) | 29.8 - 36.3 | 37.9 (293) | 34.5 - 41.3 | 10.3 (208) | 9.0 - 11.7 | 26.1 (288) | 23.6 - 28.8 | 16.0 (167) | 13.9 - 18.4 |
| High | 60.7 (3,497) | 59.4 - 61.9 | 46.9 (384) | 43.5 - 50.3 | 46.1 (357) | 42.6 - 49.7 | 76.2 (1,546) | 74.3 - 78.0 | 54.0 (595) | 51.0 - 56.9 | 59.1 (615) | 56.1 - 62.0 |
| Ethnicity |  |  |  |  |  |  |  |  |  |  |  |  |
| Majority | 79.0 (4,555) | 78.0 - 80.1 | 79.7 (653) | 76.8 - 82.3 | 73.3 (567) | 70.0 - 76.3 | 82.0 (1,663) | 80.3 - 83.6 | 87.1 (960) | 85.0 - 89.0 | 68.4 (712) | 65.5 - 71.2 |
| Minority | 21.0 (1,209) | 19.9 - 22.0 | 20.3 (166) | 17.7 - 23.2 | 26.7 (143) | 23.7 - 30.0 | 18.0 (365) | 16.4 - 19.7 | 12.9 (142) | 11.0 - 15.0 | 31.6 (329) | 28.8 - 34.5 |
| Income adequacy |  |  |  |  |  |  |  |  |  |  |  |  |
| Very difficult | 7.4 (428) | 6.8 - 8.1 | 8.4 (69) | 6.7 - 10.5 | 6.8 (53) | 5.3 - 8.9 | 8.7 (176) | 7.5 - 10.0 | 5.5 (61) | 4.3 - 7.1 | 6.6 (69) | 5.3 - 8.3 |
| Difficult | 22.5 (1,297) | 21.4 - 23.6 | 19.2 (157) | 16.6 - 22.0 | 18.0 (139) | 15.4 - 20.8 | 29.2 (592) | 27.3 - 31.2 | 21.7 (239) | 19.4 - 24.2 | 16.3 (170) | 14.2 - 18.7 |
| Neither easy nor difficult | 34.1 (1,968) | 32.9 - 35.4 | 31.9 (261) | 28.8 - 35.1 | 34.5 (267) | 31.2 - 37.9 | 38.9 (789) | 36.8 - 41.0 | 31.5 (347) | 28.8 - 34.3 | 29.2 (304) | 26.5 - 32.0 |
| Easy | 24.4 (1,406) | 23.3 - 25.5 | 27.1 (222) | 24.2 - 30.3 | 25.7 (199) | 22.8 - 28.9 | 18.9 (384) | 17.3 - 20.7 | 28.7 (316) | 26.1 - 31.4 | 27.4 (285) | 24.8 - 30.2 |
| Very easy | 11.5 (665) | 10.7 - 12.4 | 13.4 (110) | 11.3 - 15.9 | 15.0 (116) | 12.6 - 17.7 | 4.3 (87) | 3.5 - 5.3 | 12.6 (139) | 10.8 - 14.7 | 20.5 (213) | 18.1 - 23.0 |
| CI: Confidence Interval | | | | | | | | | | | | |

Supplementary Table 3. Binary Logistic Regression for the interaction between country and parental exposure and purchase request and purchase outcomes (n=5,764)

| **Outcome** | **AOR** | **CI (95%)** | **p** | **AOR** | **CI (95%)** | **p** | **AOR** | | **CI (95%)** | **p** |  |
| --- | --- | --- | --- | --- | --- | --- | --- | --- | --- | --- | --- |
|  | **Child ownership of Happy Meal or fast-food toys** | | | **Child ownership of branded merchandise with logos for unhealthy food products** | | | | **Child request for unhealthy food with licensed characters** | | | |
| **Number of locations of parental exposure** | 1.04 | 0.97 - 1.11 | 0.29 | 1.21 | 1.11 - 1.31 | 0.00* | 1.24 | | 1.14 - 1.34 | 0.00* |  |
| **Country*exposure to unhealthy food marketing** |  |  |  |  |  |  |  | |  |  |  |
| *Australia vs Canada* | 1.16 | 1.05 - 1.28 | 0.00* | 1.05 | 0.94 - 1.17 | 0.42 | 1.06 | | 0.95 - 1.18 | 0.27 |  |
| *United Kingdom vs Canada* | 1.10 | 1.00 - 1.21 | 0.06 | 1.03 | 0.93 - 1.15 | 0.54 | 1.09 | | 0.97 - 1.21 | 0.14 |  |
| *United States vs Canada* | 1.11 | 1.02 - 1.22 | 0.02* | 0.94 | 0.85 - 1.04 | 0.22 | 0.98 | | 0.89 - 1.08 | 0.69 |  |
| *Mexico vs Canada* | 1.01 | 0.93 - 1.10 | 0.78 | 0.88 | 0.81 - 0.97 | 0.01* | 0.91 | | 0.83 - 1.00 | 0.04* |  |
| *United Kingdom vs Australia* | 0.95 | 0.86 - 1.04 | 0.27 | 0.99 | 0.89 - 1.09 | 0.82 | 1.02 | | 0.92 - 1.13 | 0.67 |  |
| *United States vs Australia* | 0.96 | 0.87 - 1.05 | 0.37 | 0.90 | 0.82 - 0.99 | 0.03* | 0.92 | | 0.84 - 1.01 | 0.09 |  |
| *Mexico vs Australia* | 0.87 | 0.80 - 0.95 | 0.00* | 0.85 | 0.78 - 0.92 | 0.00* | 0.86 | | 0.79 - 0.93 | 0.00* |  |
| *United States vs United Kingdom* | 1.01 | 0.92 - 1.11 | 0.78 | 0.91 | 0.83 - 0.99 | 0.04* | 0.90 | | 0.82 - 0.99 | 0.03* |  |
| *Mexico vs United Kingdom* | 0.92 | 0.85 - 1.00 | 0.05 | 0.86 | 0.79 - 0.93 | 0.00* | 0.84 | | 0.77 - 0.91 | 0.00* |  |
| *Mexico vs United States* | 0.91 | 0.84 - 0.98 | 0.01* | 0.94 | 0.88 - 1.00 | 0.07 | 0.93 | | 0.87 - 1.00 | 0.05 |  |
|  | **Child request for unhealthy food with spokes-characters** | | | **Parental purchase of unhealthy food products with licensed characters** | | | | **Parental purchase of unhealthy food products with spokes-characters** | | | |
| **Number of locations of parental exposure** | 1.26 | 1.16 - 1.37 | 0.00* | 1.15 | 1.07 - 1.23 | 0.00* | 1.17 | | 1.08 - 1.27 | 0.00* |  |
| **Country*exposure to unhealthy food marketing** |  |  |  |  |  |  |  | |  |  |  |
| *Australia vs Canada* | 1.01 | 0.91 - 1.13 | 0.80 | 1.09 | 0.98 - 1.20 | 0.10 | 1.04 | | 0.94 - 1.16 | 0.43 |  |
| *United Kingdom vs Canada* | 1.05 | 0.94 - 1.17 | 0.38 | 1.10 | 0.99 - 1.22 | 0.09 | 1.07 | | 0.96 - 1.19 | 0.24 |  |
| *United States vs Canada* | 0.99 | 0.89 - 1.10 | 0.82 | 1.02 | 0.93 - 1.12 | 0.65 | 1.02 | | 0.92 - 1.12 | 0.72 |  |
| *Mexico vs Canada* | 0.89 | 0.81 - 0.98 | 0.01* | 0.91 | 0.84 - 0.99 | 0.03* | 0.93 | | 0.85 - 1.01 | 0.08 |  |
| *United Kingdom vs Australia* | 1.04 | 0.94 - 1.14 | 0.50 | 1.01 | 0.91 - 1.12 | 0.85 | 1.02 | | 0.93 - 1.13 | 0.64 |  |
| *United States vs Australia* | 0.97 | 0.88 - 1.07 | 0.60 | 0.94 | 0.86 - 1.03 | 0.18 | 0.98 | | 0.89 - 1.07 | 0.61 |  |
| *Mexico vs Australia* | 0.88 | 0.81 - 0.95 | 0.00* | 0.84 | 0.78 - 0.91 | 0.00* | 0.89 | | 0.82 - 0.96 | 0.00* |  |
| *United States vs United Kingdom* | 0.94 | 0.85 - 1.04 | 0.21 | 0.93 | 0.85 - 1.03 | 0.15 | 0.95 | | 0.86 - 1.05 | 0.34 |  |
| *Mexico vs United Kingdom* | 0.85 | 0.78 - 0.92 | 0.00* | 0.83 | 0.77 - 0.91 | 0.00* | 0.87 | | 0.79 - 0.94 | 0.00* |  |
| *Mexico vs United States* | 0.90 | 0.83 - 0.97 | 0.01* | 0.89 | 0.84 - 0.96 | 0.00* | 0.91 | | 0.85 - 0.98 | 0.01* |  |

*AOR: Adjusted Odds Ratio; CI: Confidence Interval; p: p-value; *p-value considered significant (p<0.05) according to Benjamini-Hochberg method; adjusted for country, the presence of children aged 5 years and under, 6 to 12 years and 12-17 years, perceived income adequacy and parental sex, age, ethnicity, and education. Reference country presented second.*

Supplementary Table 4. Generalized Ordinal Logistic Regression with interaction between country and parental exposure and intake of unhealthy food and drink products during the week (n=5,764)

| **Predictor** | **AOR** | **CI (95%)** | **p** | **AOR** | **CI (95%)** | **p** | **AOR** | **CI (95%)** | **p** | |
| --- | --- | --- | --- | --- | --- | --- | --- | --- | --- | --- |
|  | **Sugary drinks** | | | **Fast-food** | | | **Sugary cereals** | | | |
| **Moderate or high consumption (low consumption as reference)** |  |  |  |  |  |  |  |  |  | |
| **Number of locations of parental exposure** | 0.93 | 0.87 - 1.01 | 0.07 | 0.94 | 0.87 - 1.00 | 0.06 | 0.97 | 0.90 - 1.04 | 0.38 | |
| **Country*exposure to unhealthy food marketing** |  |  |  |  |  |  |  |  |  | |
| *Australia vs Canada* | 1.01 | 0.91 - 1.11 | 0.91 | 0.99 | 0.91 - 1.09 | 0.86 | 0.96 | 0.88 - 1.06 | 0.44 | |
| *United Kingdom vs Canada* | 0.96 | 0.87 - 1.06 | 0.44 | 0.95 | 0.86 - 1.05 | 0.30 | 0.95 | 0.86 - 1.04 | 0.27 | |
| *United States vs Canada* | 1.04 | 0.94 - 1.14 | 0.45 | 0.96 | 0.87 - 1.06 | 0.44 | 1.01 | 0.91 - 1.11 | 0.88 | |
| *Mexico vs Canada* | 1.06 | 0.98 - 1.16 | 0.15 | 1.05 | 0.97 - 1.14 | 0.20 | 1.00 | 0.92 - 1.10 | 0.92 | |
| *United Kingdom vs Australia* | 0.96 | 0.87 - 1.05 | 0.33 | 0.96 | 0.87 - 1.05 | 0.35 | 0.98 | 0.89 - 1.08 | 0.70 | |
| *United States vs Australia* | 1.03 | 0.95 - 1.12 | 0.47 | 0.97 | 0.88 - 1.07 | 0.50 | 1.05 | 0.96 - 1.14 | 0.33 | |
| *Mexico vs Australia* | 1.06 | 0.98 - 1.14 | 0.13 | 1.06 | 0.99 - 1.14 | 0.09 | 1.04 | 0.96 - 1.13 | 0.31 | |
| *United States vs United Kingdom* | 1.08 | 0.99 - 1.18 | 0.09 | 1.01 | 0.91 - 1.12 | 0.84 | 1.07 | 0.97 - 1.17 | 0.19 | |
| *Mexico vs United Kingdom* | 1.11 | 1.02 - 1.20 | 0.01* | 1.11 | 1.03 - 1.20 | 0.01* | 1.06 | 0.97 - 1.16 | 0.17 | |
| *Mexico vs United States* | 1.03 | 0.96 - 1.10 | 0.48 | 1.10 | 1.01 - 1.19 | 0.03* | 1.00 | 0.92 - 1.08 | 0.95 | |
| **High consumption (low and moderate consumption as reference)** |  |  |  |  |  |  |  |  |  | |
| **Number of locations of parental exposure** | 1.00 | 0.90 - 1.64 | 0.96 | 0.97 | 0.85 - 1.11 | 0.64 | 0.95 | 0.86 - 1.04 | 0.27 | |
| **Country*exposure to unhealthy food marketing** |  |  |  |  |  |  |  |  |  | |
| *Australia vs Canada* | 0.95 | 0.83 - 0.00 | 0.40 | 0.98 | 0.84 - 1.13 | 0.75 | 0.97 | 0.86 - 1.09 | 0.63 | |
| *United Kingdom vs Canada* | 0.96 | 0.84 - 1.08 | 0.56 | 0.91 | 0.77 - 1.06 | 0.23 | 0.95 | 0.84 - 1.07 | 0.38 | |
| *United States vs Canada* | 0.93 | 0.83 - 1.10 | 0.26 | 0.90 | 0.78 - 1.05 | 0.20 | 0.98 | 0.88 - 1.10 | 0.77 | |
| *Mexico vs Canada* | 1.03 | 0.92 - 1.05 | 0.59 | 1.06 | 0.90 - 1.24 | 0.48 | 1.05 | 0.95 - 1.16 | 0.37 | |
| *United Kingdom vs Australia* | 1.02 | 0.92 - 1.16 | 0.74 | 0.93 | 0.83 - 1.04 | 0.22 | 0.97 | 0.88 - 1.08 | 0.64 | |
| *United States vs Australia* | 0.99 | 0.90 - 1.13 | 0.77 | 0.93 | 0.84 - 1.03 | 0.15 | 1.01 | 0.92 - 1.11 | 0.80 | |
| *Mexico vs Australia* | 1.09 | 1.00 - 1.08 | 0.04* | 1.09 | 0.97 - 1.21 | 0.15 | 1.08 | 0.99 - 1.17 | 0.07 | |
| *United States vs United Kingdom* | 0.97 | 0.89 - 1.19 | 0.52 | 1.00 | 0.89 - 1.11 | 0.95 | 1.04 | 0.94 - 1.15 | 0.46 | |
| *Mexico vs United Kingdom* | 1.07 | 0.98 - 1.06 | 0.11 | 1.17 | 1.03 - 1.32 | 0.01* | 1.11 | 1.01 - 1.21 | 0.03* | |
| *Mexico vs United States* | 1.11 | 1.03 - 1.17 | 0.01* | 1.17 | 1.05 - 1.31 | 0.01* | 1.07 | 0.99 - 1.15 | 0.10 | |
|  | **Snacks** | | | **Dessert** | | | **Candy** | | | |
| **Moderate or high consumption (low consumption as reference)** |  |  |  |  |  |  |  |  |  | |
| **Number of locations of parental exposure** | 0.97 | 0.90 - 1.04 | 0.40 | 0.98 | 0.92 - 1.05 | 0.59 | 0.94 | 0.88 - 1.01 | 0.08 | |
| **Country*exposure to unhealthy food marketing** |  |  |  |  |  |  |  |  |  | |
| *Australia vs Canada* | 0.93 | 0.83 - 1.03 | 0.18 | 0.96 | 0.87 - 1.06 | 0.43 | 0.99 | 0.90 - 1.09 | 0.89 | |
| *United Kingdom vs Canada* | 0.99 | 0.88 - 1.11 | 0.85 | 1.02 | 0.92 - 1.13 | 0.67 | 1.09 | 0.99 - 1.21 | 0.07 | |
| *United States vs Canada* | 0.99 | 0.90 - 1.09 | 0.82 | 1.00 | 0.91 - 1.10 | 0.94 | 1.00 | 0.91 - 1.09 | 0.98 | |
| *Mexico vs Canada* | 1.04 | 0.96 - 1.13 | 0.29 | 1.02 | 0.94 - 1.11 | 0.61 | 1.06 | 0.98 - 1.15 | 0.16 | |
| *United Kingdom vs Australia* | 1.07 | 0.95 - 1.20 | 0.30 | 1.07 | 0.96 - 1.18 | 0.24 | 1.10 | 1.00 - 1.22 | 0.05 | |
| *United States vs Australia* | 1.07 | 0.96 - 1.18 | 0.21 | 1.04 | 0.94 - 1.14 | 0.45 | 1.01 | 0.92 - 1.10 | 0.90 | |
| *Mexico vs Australia* | 1.13 | 1.03 - 1.23 | 0.01* | 1.06 | 0.98 - 1.16 | 0.15 | 1.07 | 0.98 - 1.15 | 0.12 | |
| *United States vs United Kingdom* | 1.00 | 0.90 - 1.11 | 0.99 | 0.97 | 0.88 - 1.08 | 0.61 | 0.91 | 0.83 - 1.00 | 0.05 | |
| *Mexico vs United Kingdom* | 1.06 | 0.96 - 1.16 | 0.26 | 1.00 | 0.92 - 1.09 | 0.97 | 0.97 | 0.89 - 1.05 | 0.42 | |
| *Mexico vs United States* | 1.06 | 0.98 - 1.13 | 0.14 | 1.02 | 0.95 - 1.11 | 0.53 | 1.06 | 0.99 - 1.14 | 0.11 | |
| **High consumption (low and moderate consumption as reference)** |  |  |  |  |  |  |  |  |  | |
| **Number of locations of parental exposure** | 0.98 | 0.90 - 1.07 | 0.73 | 1.05 | 0.96 - 1.15 | 0.26 | 0.95 | 0.85 - 1.05 | 0.30 | |
| **Country*exposure to unhealthy food marketing** |  |  |  |  |  |  |  |  |  | |
| *Australia vs Canada* | 0.94 | 0.84 - 1.04 | 0.24 | 0.90 | 0.80 - 1.00 | 0.06 | 1.03 | 0.90 - 1.18 | 0.67 | |
| *United Kingdom vs Canada* | 0.98 | 0.88 - 1.10 | 0.76 | 0.87 | 0.78 - 0.98 | 0.02* | 0.95 | 0.83 - 1.08 | 0.44 | |
| *United States vs Canada* | 0.90 | 0.81 - 1.00 | 0.05* | 0.85 | 0.76 - 0.95 | 0.01* | 0.95 | 0.84 - 1.08 | 0.45 | |
| *Mexico vs Canada* | 1.06 | 0.95 - 1.18 | 0.33 | 0.97 | 0.87 - 1.07 | 0.52 | 1.09 | 0.96 - 1.23 | 0.17 | |
| *United Kingdom vs Australia* | 1.05 | 0.95 - 1.16 | 0.33 | 0.97 | 0.88 - 1.08 | 0.61 | 0.92 | 0.82 - 1.03 | 0.15 | |
| *United States vs Australia* | 0.96 | 0.88 - 1.05 | 0.38 | 0.95 | 0.86 - 1.05 | 0.28 | 0.93 | 0.84 - 1.02 | 0.13 | |
| *Mexico vs Australia* | 1.13 | 1.03 - 1.24 | 0.01* | 1.08 | 0.99 - 1.17 | 0.09 | 1.06 | 0.96 - 1.17 | 0.27 | |
| *United States vs United Kingdom* | 0.91 | 0.83 - 1.00 | 0.06 | 0.97 | 0.87 - 1.08 | 0.59 | 1.01 | 0.91 - 1.11 | 0.91 | |
| *Mexico vs United Kingdom* | 1.07 | 0.98 - 1.18 | 0.15 | 1.11 | 1.01 - 1.21 | 0.03* | 1.15 | 1.04 - 1.27 | 0.01* | |
| *Mexico vs United States* | 1.17 | 1.07 - 1.29 | 0.00* | 1.14 | 1.04 - 1.25 | 0.01* | 1.14 | 1.05 - 1.24 | 0.00* | |
| *AOR: Adjusted Odds Ratio; CI: Confidence Interval; p: p-value; *p-value considered significant (p<0.05) according to Benjamini-Hochberg method; adjusted for country, the presence of children aged 5 years and under, 6 to 12 years and 12-17 years, perceived income adequacy and parental sex, age, ethnicity, and education. Reference country presented second.* | | | | | | | | | |  |
